# Raised Amylase as a Potential Marker of Vascular Dysfunction and Inflammation in Fabry Disease

**DOI:** 10.1002/jmd2.70122

**Published:** 2026-11-09

**Authors:** Simona D'Amore, Ma Cabides, Uma Ramaswami

**Affiliations:** ^1^ Lysosomal Storage Disorders Unit Royal Free Hospital NHS Foundation Trust London UK; ^2^ University College London London UK

**Keywords:** amylase, Fabry disease, inflammation, lipoprotein(a), lysosomal storage disorders, pancreas

## Abstract

Fabry disease (FD) is a X‐linked disorder caused by deficiency of alpha‐galactosidase A (AGAL), leading to progressive accumulation of globotriaosylceramide (Gb3) and globotriaosylsphingosine (lyso‐Gb3). While renal, cardiac and cerebrovascular complications are well described, pancreatic involvement remains poorly characterised. We retrospectively evaluated the clinical and biochemical features of FD patients with elevated serum amylase levels and explored associations with disease severity, treatment status, inflammation and vascular dysfunction. Sixty‐two patients (35 males, 27 females; median age 53.9 years [IQR 42.1–64.5]) showed hyperamylasaemia (> 100 U/L). None had known pancreatic disease or significant alcohol intake. Median amylase levels were 114.5 U/L (IQR 104–137). Seven patients were obese (body mass index ≥ 30 kg/m^2^). No significant differences in amylase levels were observed according to sex, genotype, treatment status or renal/cerebrovascular involvement. However, patients with cardiac involvement had higher amylase levels than those without cardiac manifestations (median 128 [IQR 104–149] vs. 113 U/L [IQR 104.3–125]; *p* = 0.039). Amylase levels were not associated with AGAL activity, lyso‐Gb3 concentrations, renal function, liver function, d‐dimer, lactate dehydrogenase or lipid profile. Significant correlations were observed with age (*r* = 0.292; *p* = 0.021), body mass index (*r* = −0.382; *p* = 0.003) and lipoprotein(a) levels (*r* = 0.413; *p* = 0.004). Notably, elevated amylase occurred in the absence of clinically evident pancreatic disease, significant alcohol consumption or liver dysfunction. These findings suggest that hyperamylasaemia may represent an underrecognised biochemical feature of FD, potentially reflecting vascular and inflammatory mechanisms rather than overt pancreatic disease. Further studies are needed to clarify the pathophysiological significance of hyperamylasaemia in FD.

## Take‐Home Message

Elevated serum amylase can occur in Fabry disease in the absence of clinically evident pancreatic damage and may be linked to underlying microvascular and inflammatory mechanisms.

## 1 Introduction

Fabry disease (FD, OMIM 301500) is a rare X‐linked lysosomal storage disorder (LSD) caused by pathogenic variants in the *GLA* gene, located on the long arm of chromosome X at Xq22.1, resulting in deficient or absent activity of the alpha‐galactosidase A (AGAL) enzyme [1, 2]. The lack of AGAL activity leads to the progressive intracellular accumulation of glycosphingolipids—globotriaosylceramide (Gb3) and its deacylated derivative globotriaosylsphingosine (lyso‐Gb3)—within lysosomes of multiple cell types, including vascular endothelial cells, smooth muscle cells, cardiomyocytes, renal cells and nerve cells [1, 2]. The progressive accumulation of Gb3 and lyso‐Gb3 causes lysosomal dysfunction, chronic inflammation, oxidative stress, vascular smooth muscle cell proliferation, cellular injury and death. These processes ultimately impair organ perfusion and promote fibrosis, leading to irreversible damage in several organs, such as kidneys, heart and brain [3, 4].

The clinical phenotype of FD is highly heterogeneous, ranging from the classical early‐onset form (characterised by neuropathic pain, angiokeratomas, hypohidrosis, cornea verticillata and with predominant renal involvement) to late‐onset variants (with predominant, although not exclusively, cardiac involvement) [5, 6].

Beyond these well‐recognised manifestations, gastrointestinal (GI) involvement is commonly observed in FD, affecting up to 50%–70% of patients and significantly impacting patients' quality of life [7, 8]. Non‐specific GI symptoms appear early in the disease course and include severe postprandial abdominal pain and intermittent or chronic diarrhoea—particularly after fatty meals—constipation, bloating, recurrent nausea and vomiting, which mimic irritable bowel syndrome (IBS) and are thought to arise from a combination of autonomic neuropathy, smooth muscle dysfunction, vascular insufficiency and tissue inflammation related to glycosphingolipid accumulation of the GI tract [9, 10, 11, 12, 13]. These pathological mechanisms result in alterations such as accelerated intestinal transit, impaired peristalsis with subsequent small intestine bacterial overgrowth, nutrient malabsorption, gastroparesis and ischemic or neuropathic injury, which contribute to the GI manifestations associated with FD [11, 12, 13]. Patients with GI symptoms may benefit from a diet low in short‐chain fermentable carbohydrates (low‐FODMAP diet), and from avoiding certain foods such as caffeine, alcohol, insoluble fibre, fat, spices and spicy foods [14]. The use of pancreatic enzymes has also been observed improving GI symptoms in FD patients [10].

In contrast, very little is known regarding pancreatic involvement in FD and available findings are inconclusive [15, 16]. To date, only one study has evaluated the exocrine pancreatic function in FD, reporting normal faecal elastase values and no evidence of exocrine pancreatic insufficiency in all analysed patients; however, the study cohort was relatively small (28 FD patients), and most patients (17/28; 63%) were receiving enzyme replacement therapy (ERT) at the time of assessment [15]. The pancreas, similarly to other highly vascularised organs, may be prone to glycosphingolipid accumulation within endothelial and smooth muscle cells, potentially resulting in subclinical ischemia and chronic inflammatory changes as observed in other tissues [17]. This mechanism may mirror the metabolic dysfunction and inflammatory pathways (‘metainflammation’) described in more common glycolipid metabolic disorders, such as obesity, diabetes and metabolic syndrome, in which abnormalities in glucose and lipid metabolism contribute to tissue injury and systemic inflammation [18]. Nevertheless, the clinical significance of pancreatic involvement in FD remains poorly characterised, and pancreatic biomarkers have not been systematically investigated in FD populations.

Amylase is a calcium‐dependent hydrolase that circulates as two major isoenzymes, pancreatic (P‐type) and salivary (S‐type), both existing as monomeric proteins whose primary function is the hydrolysis of complex carbohydrates into simpler sugars [19]. P‐type amylase is synthesised by pancreatic acinar cells and secreted into the intestinal lumen through the pancreatic ductal system, whereas S‐type amylase is produced predominantly by the salivary glands, with smaller amounts also synthesised by the testes, ovaries, fallopian tubes, lungs, striated muscles and adipose tissue [19]. In the circulation, amylase is present predominantly in its free form, although a small proportion may bind to immunoglobulins—most commonly IgA, followed by IgG—or other plasma proteins, forming macroamylase. This benign condition has been associated with autoimmune disease, malignancies, cardiovascular disease, diabetes mellitus and malabsorptive disorders and results in reduced renal clearance with consequent hyperamylasaemia [19].

Beyond its physiological role in digestion, serum amylase is a widely available biomarker routinely used in the evaluation of pancreatic disorders [19]. Although serum amylase has been associated with metabolic disorders and alterations in lipid profiles [20, 21], no direct biochemical interactions with circulating lipoproteins have been demonstrated, suggesting that these associations reflect shared metabolic or inflammatory pathways rather than a causal relationship. However, although sensitive, its diagnostic accuracy is limited by moderate specificity, as serum levels may be influenced by several non‐pancreatic factors, including impaired renal clearance and subsequent decreased amylase clearance, liver dysfunction, salivary gland disorders, alcohol consumption, heart failure and systemic inflammatory conditions [22, 23]. Interestingly, mild hyperamylasaemia is frequently encountered in clinical practice and may occur in the absence of clinically evident pancreatic disease [24]. To date, hyperamylasaemia has not been systematically reported in other LSD; however, many LSDs are associated with hepatic and renal dysfunction [25], and because both organs contribute to amylase clearance, impaired liver and/or kidney function could reduce amylase elimination, leading to elevated serum concentrations. Hence, in the context of multisystem disorders such as FD, the interpretation of hyperamylasaemia could therefore be challenging as it may reflect extra‐pancreatic mechanisms.

In this study, we aimed to characterise the clinical and biochemical profile of FD patients attending the Lysosomal Storage Disorders Unit (LSDU) at a specialist centre who showed increased amylase levels (defined as amylase greater than 100 U/L), and to explore potential associations with sex, genotype, disease severity, treatment status, markers of inflammation and vascular dysfunction.

## 2 Methods

### 2.1 Study Design and Population

This was a retrospective observational analysis conducted at a single LSD referral centre. Adult patients with a confirmed clinical, biochemical and genetic diagnosis of FD and who had at least one recorded serum amylase measurement were included in the analysis. The patients included those diagnosed with classic and non‐classic disease variants [5].

### 2.2 Definition of Hyperamylasaemia

Elevated amylase was defined as serum amylase > 100 U/L, based on local laboratory reference ranges. Potential confounding factors associated with hyperamylasaemia were assessed, including known pancreatic or liver disease, gallbladder disease, significant alcohol consumption and obesity.

### 2.3 Data Collection

Clinical and biochemical data were extracted from electronic medical records, including demographics (age, sex), body mass index (BMI categories were defined according to WHO criteria as follows: healthy weight 18.5–24.9 kg/m^2^, overweight 25.0–29.9 kg/m^2^, Obesity Class I 30.0–34.9 kg/m^2^, Obesity Class II 35.0–39.9 kg/m^2^ and severe obesity (Class III) ≥ 40 kg/m^2^) [26], genotype, clinical history (gallstones; cerebrovascular events; cardiac events and end‐stage kidney disease), treatment status (ERT or oral chaperone therapy) and laboratory parameters, including renal and liver function tests, lipid profile with lipoprotein(a) [Lp(a)], d‐dimer and lactate dehydrogenase (LDH).

Plasma AGAL activity and lyso‐Gb3—a biomarker commonly used for the diagnosis and monitoring of FD [27, 28]—were measured as part of routine clinical care. Reference ranges for AGAL activity were as follows: unaffected individuals, 4.0–21.9 nmol/h/mL; hemizygous FD patients, 0–0.9 nmol/h/mL and heterozygous FD patients, 0.22–7.4 nmol/h/mL. The reference range for lyso‐Gb3 was 0–1.8 ng/mL. Reference intervals were provided by Camelia Botnar Laboratories.

### 2.4 Statistical Analysis

Continuous variables are presented as median and interquartile range (IQR), categorical variables are presented as number and percentage (%). *t*‐Test was applied for group comparisons. Correlations were assessed using Pearson correlation coefficient. *p* < 0.05 was considered statistically significant. All analyses were conducted using NCSS 2023 software (version 23.0.2; Kaysville, UT, USA).

## 3 Results

### 3.1 Patient Characteristics

A total of 62 FD patients with elevated amylase were identified (35 [56.5%] males, 27 [43.5%] females), with a median age of 53.9 years (IQR 42.1–64.5). Median BMI was 23.2 (IQR 21.1–27.3), and seven (11%) patients were classified as obese. Thirty‐five (56%) patients carried non‐classic *GLA* variant, while 24 (39%) had classic *GLA* variants; genotype data were unavailable for three (5%) individuals. At the time of analysis, 30 (48%) patients were receiving ERT, and 19 (31%) were treated with oral chaperone therapy, with a median treatment duration of 5.54 years (IQR 1.25–8.74). Thirteen (21%) patients were not receiving disease‐modifying therapy (DMTs) (Table 1).

**TABLE 1:** Clinical characteristics of the study cohort.

Clinical variables	Overall
Sex, *N* (%)	
Male	35 (56.5)
Female	27 (43.5)
Age (years), median (IQR)	53.9 (42.1–64.5)
BMI (kg/m^2^), median (IQR)	23.2 (21.1–27.3)
*GLA* variant, *N* (%)	
Non‐classic variants	35 (56)
Classic variants	24 (39)
Missing	3 (5)
Treatment status, *N* (%)	
Enzyme replacement therapy	30 (48)
Oral chaperone therapy	19 (31)
Naive	13 (21)
Duration of treatment (years), median (IQR)	5.54 (1.25–8.74)
AGAL (nmol/h/mL), median (IQR)	0.35 (0.1–3.3)
Lyso‐Gb3 (ng/mL), median (IQR)	4.6 (2.2–15.05)
ALT (U/L), median (IQR)	23 (16.5–32)
GGT (U/L), median (IQR)	21.5 (14.5–33.5)
eGFR^a^ (mL/min/1.73 m^2^), median (IQR)	80 (64–90)
Total cholesterol (mmol/L), median (IQR)	4.5 (3.8–5.3)
LDL cholesterol (mmol/L), median (IQR)	2.2 (1.7–2.9)
Lp(a) (nmol/L), median (IQR)	37 (14–114)
LDH (U/L), median (IQR)	228 (189–266)
d‐dimer (ng/mL), median (IQR)	397 (306.5–556.5)
Amylase (U/L), median (IQR)	114.5 (104–137)

Abbreviations: AGAL, alpha‐galactosidase A; ALT, alanine aminotransferase; BMI, body mass index; eGFR, estimated glomerular filtration rate; GGT, gamma‐glutamyl transferase; IQR, interquartile range; LDH, lactate dehydrogenase; Lp(a), lipoprotein(a); lyso‐Gb3, globotriaosylsphingosine.

^a^ eGFR was calculated using the CKP‐EPI (Chronic Kidney Disease Epidemiology Collaboration) equation.

Regarding clinical history, one (2%) patient had gallstones; six (10%) had a history of cerebrovascular disease, including one transient ischemic attack (TIA) and five strokes; and 34 (55%) exhibited cardiac involvement, ranging from structural remodelling, such as cardiac hypertrophy, to rhythm disturbances, including atrial fibrillation. Among these, seven patients required pacemaker or implantable cardioverter‐defibrillator placement, and one underwent heart transplantation. Finally, 10 (16%) patients developed end‐stage kidney disease, requiring dialysis in one case and kidney transplantation in four. Two (3%) patients had died at the time of writing. Patients were not receiving any medication that could contribute to elevated amylase levels, such as azathioprine, steroids or other drugs. No patients were on pancreatic supplements.

### 3.2 AGAL and lyso‐Gb3 Levels

In the overall cohort, median AGAL was 0.35 nmol/h/mL (IQR 0.1–3.3), with lower levels in males (median 0.1, IQR 0.1–0.3) than females (median 3.7; IQR 2.8–5.3) and in those receiving DMTs (median 0.2; IQR 0.1–2.8) than naïve patients (median 3.8; IQR 1.6–3.8), in patients with classic *GLA* variants (median 0.3; IQR 0.1–3.2) than those with non‐classic *GLA* variant (median 0.5; IQR 0.1–3.5). Overall, median lyso‐Gb3 values were 4.6 ng/mL (IQR 2.2–15.05), with higher levels in males (median 10.6; IQR 3.1–33.73) than females (median 2.7; IQR 1.5–5.8), in those on DMTs (median 7.2, IQR 3–20.2) than naïve patients (median 1.8; IQR 1.4–4.6) and in individuals with classic *GLA* variants (median 10.7; IQR 7.9–33.7) than those with non‐classic *GLA* variant (median 2.9; IQR 1–5‐5.0) (Table 1).

### 3.3 Biochemistry Profile

In the overall cohort, median alanine aminotransferase (ALT) levels were 23 U/L (IQR 16.5–32), median gamma‐glutamyl transferase (GGT) values were 21.5 U/L (IQR 14.5–33.5) and median estimated glomerular filtration rate (eGFR) was 80 mL/min/1.73 m^2^ (IQR 64–90). Median total cholesterol was 4.5 mmol/L (IQR 3.8–5.3), low‐density lipoprotein (LDL) cholesterol 2.2 mmol/L (IQR 1.7–2.9), triglycerides 1.3 mmol/L (IQR 0.9–1.8) and Lp(a) 37 nmol/L (IQR 14–114). Median LDH levels were 228 U/L (IQR 189–266), while median d‐dimer levels were 397 ng/mL (IQR 306.5–556.5) (Table 1).

### 3.4 Amylase Levels

Overall, the median amylase level was 114.5 U/L (IQR 104–137). Amylase values were similar between males (median 120 U/L, IQR 104–137) and females (median 114 U/L, IQR 104–137; *p* = 0.798), between patients with non‐classic *GLA* variants (median 113 U/L, IQR 104–130) and those with classic *GLA* variants (median 129.5 U/L, IQR 106–153; *p* = 0.172), and between patients receiving DMTs (median 116 U/L, IQR 104–137) and treatment‐naïve patients (median 113 U/L, IQR 107–134; *p* = 0.666).

Although not statistically significant, higher values of amylase were observed in patients with renal (median 133 U/L, IQR 114.8–156.3) and cerebrovascular involvement (median 120 U/L, IQR 102.5–151‐3) compared with those without renal (median 114.5 U/L, IQR 104.8–134.8; *p* = 0.112) and cerebrovascular manifestations (median 113 U/L, IQR 104–132; *p* = 0.999). A statistically significant difference was observed in patients with cardiac involvement, who showed higher amylase levels (median 128 U/L, IQR 104–149), compared with those without cardiac involvement (median 113 U/L, IQR 104.3–125; *p* = 0.039) (Figure 1).

**FIGURE 1:**
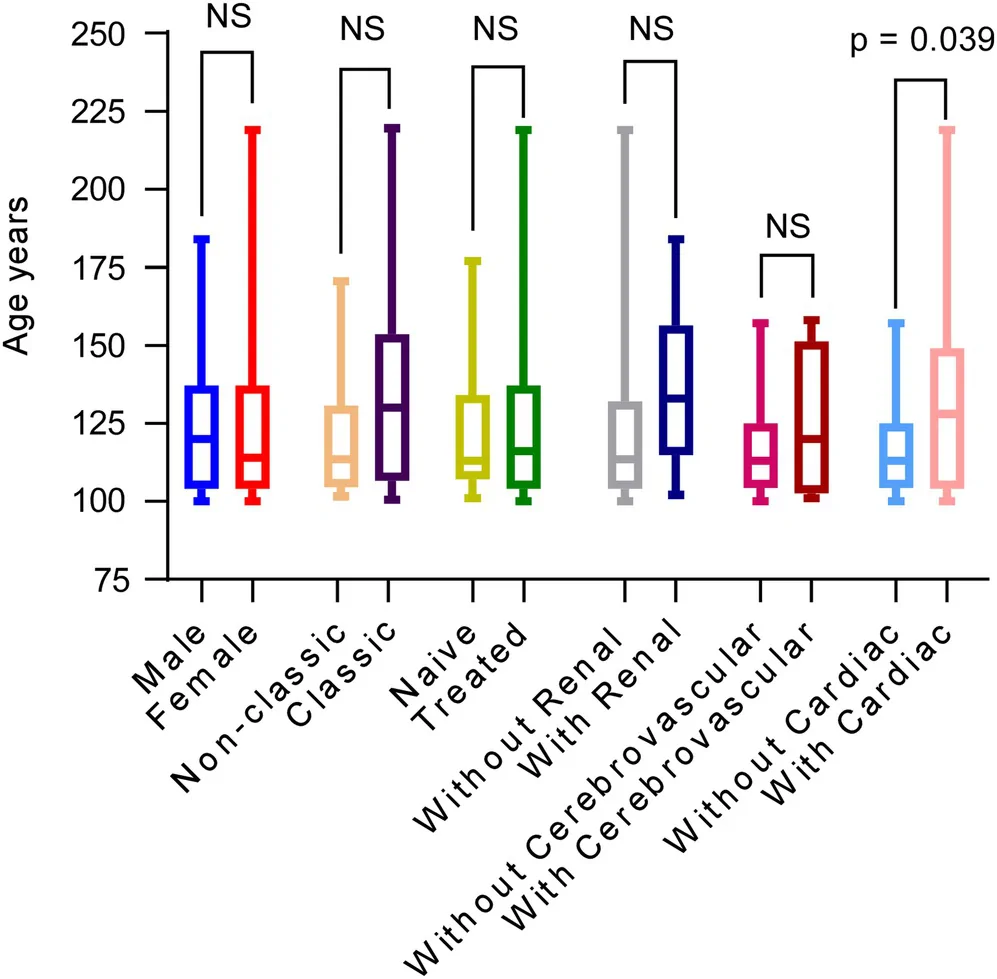
Amylase levels in patients with Fabry disease by sex, genotype, treatment status and renal, cerebrovascular and cardiac involvement. No differences in amylase values were observed between males and females; between patients with non‐classic *GLA* variants and those with classic *GLA* variants; between patients receiving disease‐modifying therapy and treatment‐naïve individuals; or according to renal or cerebrovascular involvement. In contrast, patients with cardiac involvement showed significantly higher amylase levels (median 128 U/L, IQR 104–149) compared with those without cardiac involvement (median 113 U/L, IQR 104.3–125; *p* = 0.039).

### 3.5 Correlation Analyses

Amylase levels showed a modest but statistically significant correlation with age (*r* = 0.292; *p* = 0.021), as well as stronger correlations with BMI (*r* = −0.382; *p* = 0.003) and Lp(a) levels (*r* = 0.413; *p* = 0.004), Figure 2. No significant associations were observed between amylase levels and AGAL activity, lyso‐Gb3 concentrations, renal or liver function parameters, with other lipid profile parameters, d‐dimer or LDH, except for a trend toward significance for GGT (*r* = 0.237; *p* = 0.068).

**FIGURE 2:**
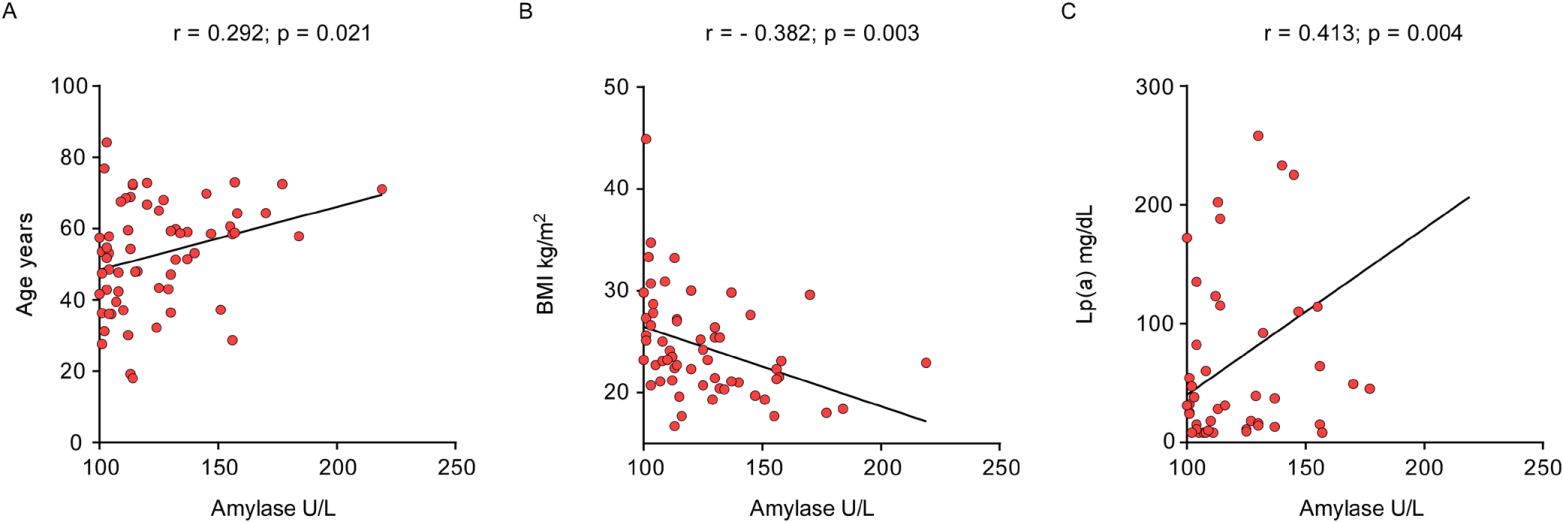
Correlations between amylase levels and clinical and biochemical parameters in patients with Fabry disease. Amylase levels showed statistically significant correlations with (A) age (*r* = 0.292; *p* = 0.021), (B) BMI (*r* = −0.382; *p* = 0.003) and (C) lipoprotein(a) levels (*r* = 0.413; *p* = 0.004).

## 4 Discussion

In this retrospective cohort of FD patients with hyperamylasaemia, we observed that mild elevations in serum amylase occurred in the absence of clinically evident pancreatic, liver, renal or alcohol‐related disease. Importantly, amylase levels were not associated with sex, genotype, treatment status, AGAL activity and lyso‐Gb3 concentrations or the presence of renal or cerebrovascular manifestations. In contrast, associations with the presence of cardiovascular manifestations and Lp(a) values were noted, suggesting that hyperamylasaemia in FD may represent an underrecognised feature potentially reflecting systemic vascular dysfunction and inflammatory mechanisms occurring in FD rather than clinically evident pancreatic disease.

To the best of our knowledge, this is the first study specifically exploring serum amylase abnormalities in FD. Previous evidence regarding pancreatic involvement in FD is limited to a small study evaluating exocrine pancreatic function through faecal elastase measurements [15]. This study did not identify clinically relevant pancreatic insufficiency; however, it included a small cohort of patients (*n* = 28), only seven of whom (25.9%) presented with abdominal complaints, potentially introducing a selection bias. In addition, most patients (17/28; 63%) were receiving ERT at the time of evaluation, including those with GI symptoms, which may have masked subtle pancreatic dysfunction [15]. In this scenario, our findings expand the current knowledge suggesting that subclinical biochemical alterations of amylase may occur even in the absence of clinically evident pancreatic disease.

To date, direct histopathological evidence of pancreatic Gb3 deposition in humans or animal models remains lacking. Nonetheless, several pathophysiological mechanisms may explain the observed hyperamylasaemia. Experimental *GLA* knockout mouse models have demonstrated widespread accumulation of Gb3 and lyso‐Gb3 across multiple visceral organs, particularly in highly vascularised tissues, together with progressive vascular dysfunction characterised by impaired endothelial relaxation and altered cellular signalling secondary to endothelial glycosphingolipid storage [29, 30, 31]. Similarly, post‐mortem evaluations in humans have confirmed extensive Gb3 accumulation in multiple organs, accompanied by histopathological abnormalities associated with chronic microvascular dysfunction, tissue ischemia and progressive organ damage that may persist despite disease‐specific treatment [32, 33, 34, 35]. The pancreas is highly vascularised; therefore, similar mechanisms of endothelial injury, microvascular ischemia and chronic low‐grade inflammation may contribute to subtle injury and/or dysregulated enzyme release, thus explaining the mild hyperamylasaemia observed in this cohort.

In line with this hypothesis, we observed a positive, statistically significant correlation between amylase levels and Lp(a) concentrations. Lp(a), a LDL‐like molecule bound to a highly glycosylated apolipoprotein(a), is a genetically driven, recognised marker of vascular dysfunction, inflammation and thrombogenicity and an established independent risk factor for atherosclerotic cardiovascular disease (ASCVD) [36, 37]. Although elevated Lp(a) is associated with an increased risk of coronary artery disease in the general population, patients with FD rarely develop obstructive coronary atherosclerosis despite significant vascular involvement. In our cohort, median Lp(a) concentrations were within the low cardiovascular risk range (< 90 nmol/L), although eight patients had values associated with increased cardiovascular risk (90–200 nmol/L) and six had markedly elevated levels (≥ 200 nmol/L), which are linked to substantially increased cardiovascular and thrombotic risk. Although hypothetical, the association observed between amylase and Lp(a) may support the hypothesis that hyperamylasaemia reflects underlying vascular and inflammatory abnormalities rather than primary pancreatic injury. Glycosphingolipid accumulation within the pancreatic microvasculature, together with additional vascular insults such as Lp(a)‐mediated inflammation driven by oxidised phospholipids, may promote endothelial dysfunction and chronic low‐grade ischaemia, ultimately resulting in subtle acinar cell injury and enzyme leakage. Such a mechanism could explain the observed association between amylase and Lp(a) despite the absence of clinically evident pancreatic disease.

Similarly, the correlation with age may reflect cumulative microvascular damage over time. Age‐related significant changes in pancreatic morphology, perfusion and function are well known and can impact both the endocrine (hormone‐producing) and exocrine (enzyme‐producing) systems, potentially resulting, respectively, in glucose intolerance and malabsorption [38]. In contrast, the negative correlation observed with BMI differs from findings typically reported in metabolic syndrome and obesity‐related pancreatic dysfunction, where higher BMI represents a risk factor for both the onset and progression of acute pancreatitis [39]. This discrepancy may be due to a distinct metabolic and vascular pathophysiological mechanism in FD compared with other common metabolic disorders.

Interestingly, FD patients with cardiac involvement exhibited significantly higher amylase levels compared with those without cardiac manifestations. Cardiac disease in FD is characterised by progressive hypertrophy, fibrosis, endothelial dysfunction and perfusion defects, leading to altered systemic haemodynamics in advanced disease stages [40]. In non‐FD patients, heart failure and altered haemodynamics are known to impair pancreatic perfusion and may lead to pancreatic enzyme elevation through venous congestion and tissue hypoxia [41]. Therefore, the higher amylase levels observed in patients with cardiac involvement may further support the hypothesis that systemic vascular dysfunction and altered haemodynamics contribute to hyperamylasaemia in FD.

Taken together, these observations support a multifactorial model in which ageing, chronic low‐grade inflammation, endothelial dysfunction, altered haemodynamic and autonomic dysfunction may collectively contribute to subtle pancreatic injury and hyperamylasaemia in FD (Figure 3).

**FIGURE 3:**
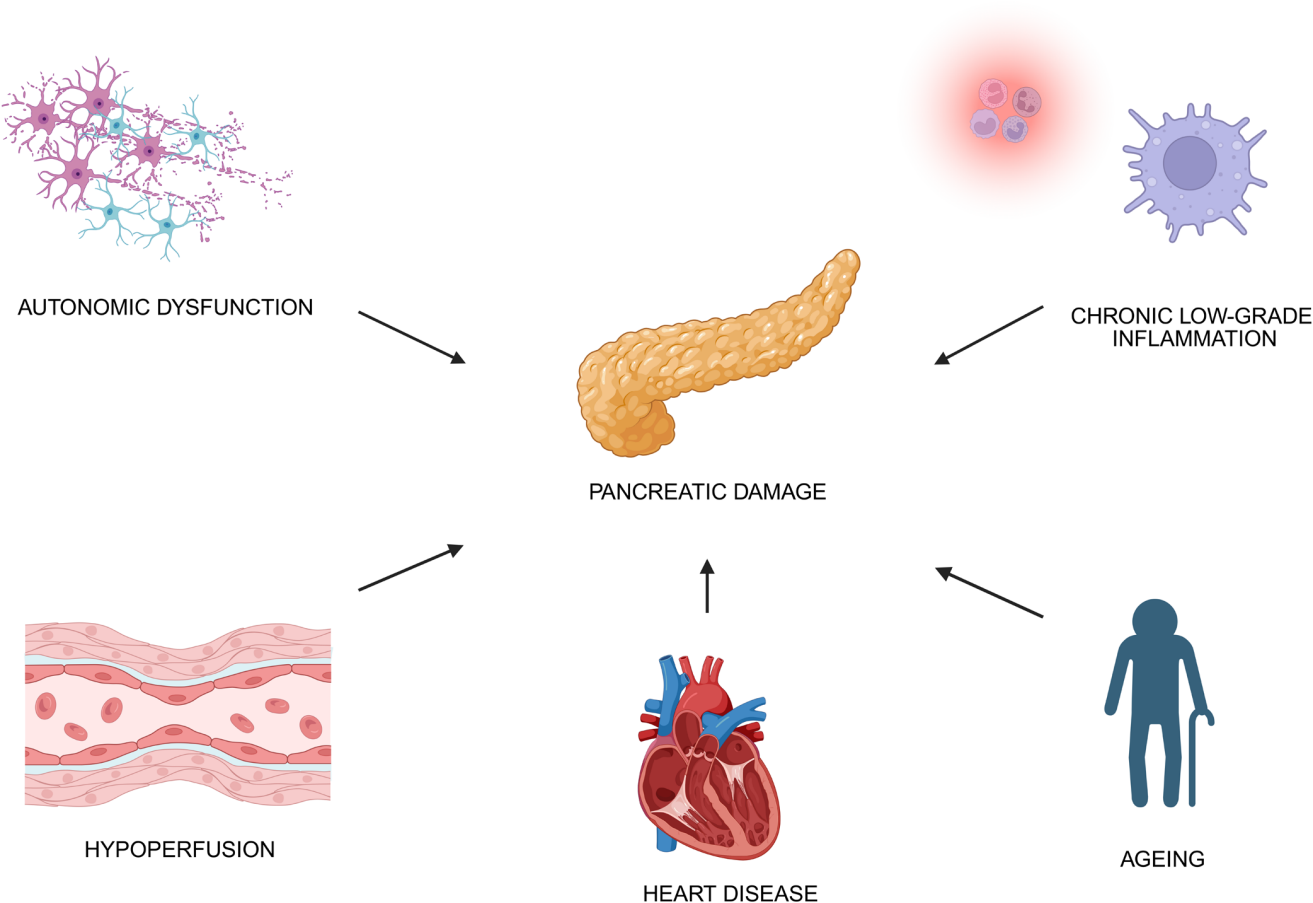
Proposed mechanisms of pancreatic damage in Fabry disease. Ageing, deranged haemodynamic, chronic low‐grade inflammation, autonomic dysfunction and heart disease could all be contributing factors of pancreatic damage in Fabry disease. Created in BioRender. https://BioRender.com/8dzc0dq.

Noteworthy, amylase levels were not associated with AGAL activity or lyso‐Gb3 concentrations. Lyso‐Gb3 is an established sensitive biomarker of disease severity and treatment response, particularly in classical FD [27, 28]. The absence of correlation in our cohort suggests that hyperamylasaemia is unlikely to be directly driven by disease severity (i.e., enzymatic deficiency severity and glycosphingolipid accumulation) and may instead reflect downstream processes. Similarly, no differences were observed according to treatment status (i.e., ERT or oral chaperone therapy vs. untreated individuals), although the interpretation of this finding is limited by the cross‐sectional and retrospective nature of the study.

Another interesting finding was the absence of significant associations between amylase and kidney function. Since amylase is partially cleared by the kidneys and reduced renal clearance is a well‐recognised cause of elevated amylase levels [42, 43], our findings exclude kidney dysfunction as the primary explanation for hyperamylasaemia in this cohort, despite the presence of advanced renal disease in a subset of patients (five patients had eGFR values less than 30 mL/min/1.73 m^2^). Nonetheless, subtle or fluctuating changes in renal function may still play a contributory role in individual patients.

Likewise, normal liver function and the exclusion of other major confounding conditions, such as significant alcohol intake, support the hypothesis that the biochemical abnormalities observed may be intrinsically related to FD.

The clinical significance of isolated hyperamylasaemia in FD remains unclear; therefore, our findings do not currently support routine pancreatic imaging or invasive investigations in asymptomatic patients but rather highlight the need for increased awareness of potential pancreatic or metabolic involvement in FD.

Our study has several limitations. First, its retrospective design and the lack of isoenzyme differentiation preclude definitive attribution of amylase elevation to pancreatic or salivary sources. Second, although we hypothesise that hyperamylasaemia may reflect vascular inflammation, the present study did not include classical inflammatory biomarkers, such as C‐reactive protein (CRP), erythrocyte sedimentation rate (ESR), cytokines profiling or endothelial activation markers. CRP and ESR were not systematically collected; however, in the subset of patients for whom these data were available, both biomarkers were within normal ranges. Consequently, the proposed inflammatory mechanism remains speculative and is supported primarily by the well‐established inflammatory pathophysiology of FD together with the observed association between Lp(a), itself recognised as a marker of vascular inflammation and endothelial dysfunction. Future studies incorporating systemic inflammatory biomarkers, vascular imaging and endothelial function assessment will be needed to validate whether hyperamylasaemia represents a surrogate marker of vascular inflammation in FD. Third, the absence of a group of FD patients with normal amylase limits our ability to define the true prevalence of this finding. Finally, the relatively limited sample size may have reduced the ability to detect potential associations between amylase and markers of disease severity. Another consideration is the potential analytical interference of glycosphingolipid accumulation with biochemical assays. However, serum amylase is routinely measured using robust enzymatic methods that have not been reported to be affected by Gb3 or lyso‐Gb3 accumulation. Moreover, the absence of systematic analytical artefacts in previous biochemical studies of FD makes assay interference an unlikely explanation for the observed findings. Nevertheless, because isoenzyme analysis and macroamylase testing were not performed, analytical or biochemical contributors cannot be completely excluded.

Despite these limitations, our study provides preliminary evidence that hyperamylasaemia may represent an underrecognised biochemical feature in FD that may reflect complex interactions between metabolic, vascular and inflammatory pathways.

Future prospective studies should aim to validate these findings in larger cohorts, incorporate detailed pancreatic assessment and explore longitudinal changes in amylase in relation to disease progression and therapeutic response.

## Author Contributions

Project conception and design: Uma Ramaswami. Data collection: Ma Cabides. Data analysis and interpretation: Uma Ramaswami, Simona D'Amore and Ma Cabides. Drafting and revision of the manuscript: Uma Ramaswami and Simona D'Amore. All authors reviewed and approved the final version of the manuscript and agreed to its submission.

## Funding

The authors have nothing to report.

## Ethics Statement

This was a service evaluation project and did not require ethical approval since enzymatic assay, genetic testing along with other investigations are part of routine clinical care.

## Consent

The authors have nothing to report.

## Conflicts of Interest

The authors declare no conflicts of interest.

## Data Availability

The data that support the findings of this study are available from the corresponding author upon reasonable request.
